# Supplementary material for: Fragment-Based Ab Initio Molecular Dynamics Simulation for Combustion
Source: Molecules. 2021 May 23;26(11):3120. doi: 10.3390/molecules26113120 (PMC8197069; doi:10.3390/molecules26113120)
Supplement: Supplementary file 1 [file molecules-26-03120-s001.zip › molecules-1171076-supplementary.pdf]

Supporting Information

Article

Fragment-Based Ab Initio Molecular Dynamics Simulation for Combustion

Liqun Cao [1], Jinzhe Zeng [2], Mingyuan Xu [1], Chih-Hao Chin [1], Tong Zhu [1,3,*] and John Z. H. Zhang [1,3,4,*]

[1] School of Chemistry and Molecular Engineering, Shanghai Engineering Research Center of Molecular Therapeutics & New Drug Development, East China Normal University, Shanghai 200062, China; caoliqun1994@163.com (L.C.); mingyuan_xu2014@163.com (M.X.); zhjin@chem.ecnu.edu.cn (C.-H.C.)
[2] Department of Chemistry and Chemical Biology, Rutgers University, Piscataway, NJ 08854, USA; jinzhe.zeng@rutgers.edu
[3] NYU-ECNU Center for Computational Chemistry, NYU Shanghai, Shanghai 200062, China
[4] Department of Chemistry, New York University, New York, NY 10003, USA
* Correspondence: tzhu@lps.ecnu.edu.cn (T.Z.); john.zhang@nyu.edu (J.Z.H.Z.); Tel.: +86-18801626247 (T.Z.); +86-2120596108 (J.Z.H.Z.)

Boundary condition with Jacobi Coordinates

In theoretical chemistry, Jacobi coordinates are usually used to reduce degrees of freedoms in the calculation of polyatomic reactions. For the N-body problem, the Jacobi Coordinates can be written as

$$\boldsymbol{r}_j = \frac{1}{m_{0j}}\sum_{k=1}^{j} m_k \boldsymbol{x}_k - \boldsymbol{x}_{j+1}\ (j = 1,2,\ldots.,N-1) \tag{1}$$

$$\boldsymbol{r}_N = \frac{1}{m_{0N}}\sum_{k=1}^{N} m_k \boldsymbol{x}_k \tag{2}$$

$$m_{0j} = \sum_{k=1}^{j} m_k \tag{3}$$

where $\boldsymbol{r}_j$ represents the relative distance between the atom $j+1$ and the center of mass of the first j atoms; m_k represents the mass of atom k; $\boldsymbol{x}_k$ represents the coordinates of atom k; $\boldsymbol{r}_N$ represents the coordinate of the center of mass of N atoms; m_{0j} represents the total mass of the first j atoms.

According to the coordinates, we can derive the velocity

$$\boldsymbol{V}_j = \frac{1}{m_{0j}}\sum_{k=1}^{j} m_k \boldsymbol{v}_k - \boldsymbol{v}_{j+1}\ (j = 1,2,\ldots.,N-1) \tag{4}$$

$$\boldsymbol{V}_C = \frac{1}{m_{0N}}\sum_{k=1}^{N} m_k \boldsymbol{v}_k \tag{5}$$

$$m_{0j} = \sum_{k=1}^{j} m_k \tag{6}$$

Correspondingly, $\boldsymbol{V}_j$ represents the relative velocity between the atom *j+1* and the center of mass of the first *j* atoms; $\boldsymbol{v}_k$ represents the velocity of atom *k*; $\boldsymbol{V}_C$ represents the velocity of the center of mass of *N* atoms.

Assume that the initial velocity is a row matrix

$$\boldsymbol{I}_k = [\boldsymbol{v_1}, \boldsymbol{v_2}, \boldsymbol{v_3}, \dots\dots, \boldsymbol{v_k}] \tag{7}$$

It can be converted to Jacobi velocity

$$\begin{bmatrix} \boldsymbol{V_1} \\ \boldsymbol{V}_2 \\ \boldsymbol{V}_3 \\ \cdot \\ \cdot \\ \cdot \\ \boldsymbol{V}_C \end{bmatrix} = \begin{bmatrix} \mathbf{1} & \mathbf{-1} & \mathbf{0} & \dots & \mathbf{0} & \mathbf{0} \\ \frac{\boldsymbol{m_1}}{\boldsymbol{m_1}+\boldsymbol{m_2}} & \frac{\boldsymbol{m_2}}{\boldsymbol{m_1}+\boldsymbol{m_2}} & \mathbf{-1} & \mathbf{0} & \dots & \mathbf{0} \\ \frac{\boldsymbol{m_1}}{\boldsymbol{m_1}+\boldsymbol{m_2}+\boldsymbol{m_3}} & \frac{\boldsymbol{m_2}}{\boldsymbol{m_1}+\boldsymbol{m_2}+\boldsymbol{m_3}} & \frac{\boldsymbol{m_3}}{\boldsymbol{m_1}+\boldsymbol{m_2}+\boldsymbol{m_3}} & \mathbf{-1} & \mathbf{0} & \mathbf{0} \\ \vdots & \vdots & \ddots & & & \vdots \\ \vdots & \vdots & & \ddots & & \vdots \\ \frac{\boldsymbol{m_1}}{\sum_{k=1}^{N}\boldsymbol{m_k}} & \frac{\boldsymbol{m_2}}{\sum_{k=1}^{N}\boldsymbol{m_k}} & \dots & \dots & \dots & \frac{\boldsymbol{m_k}}{\sum_{k=1}^{N}\boldsymbol{m_k}} \end{bmatrix} \begin{bmatrix} \boldsymbol{v_1} \\ \boldsymbol{v_2} \\ \boldsymbol{v_3} \\ \cdot \\ \cdot \\ \cdot \\ \boldsymbol{v_k} \end{bmatrix} \tag{8}$$

Formula (8) can be simplified as

$$\boldsymbol{V} = \boldsymbol{A} \times \boldsymbol{I}_k^T \tag{9}$$

We set a matrix

$$\boldsymbol{B} = \begin{bmatrix} \mathbf{1} & \mathbf{0} & \cdots & \cdots & \mathbf{0} \\ \mathbf{0} & \mathbf{1} & \mathbf{0} & \cdots & \mathbf{0} \\ \vdots & \mathbf{0} & \ddots & \mathbf{0} & \vdots \\ \vdots & \vdots & \mathbf{0} & \mathbf{1} & \mathbf{0} \\ \mathbf{0} & \cdots & \cdots & \mathbf{0} & \mathbf{-1} \end{bmatrix} \tag{10}$$

After collision, the velocity of the center of mass is reversed

$$\begin{bmatrix} \boldsymbol{V}_1 \\ \boldsymbol{V}_2 \\ \boldsymbol{V}_3 \\ \cdot \\ \cdot \\ \cdot \\ -\boldsymbol{V}_C \end{bmatrix} = \boldsymbol{B} \times \begin{bmatrix} \boldsymbol{V}_1 \\ \boldsymbol{V}_2 \\ \boldsymbol{V}_3 \\ \cdot \\ \cdot \\ \cdot \\ \boldsymbol{V}_C \end{bmatrix} \tag{11}$$

Then we transform the Jacobi velocicy back to atomic velocities in the Cartesian coordinates.

$$\begin{bmatrix} v'_1 \\ v'_2 \\ v'_3 \\ \cdot \\ \cdot \\ \cdot \\ v'_k \end{bmatrix} = A^{-1} \times \begin{bmatrix} V_1 \\ V_2 \\ V_3 \\ \cdot \\ \cdot \\ \cdot \\ -V_C \end{bmatrix} \quad (12)$$

If we set

$$I'_k = [v'_1, v'_2, v'_3, \dots \dots, v'_k] \quad (13)$$

Formula (12) can be simplified as

$$I_k'^T = A^{-1} \times B \times A \times I_k^T \quad (14)$$

Finally, we convert the atomic velocity v_k to the velocity v'_k when any atom of the molecule hits the boundary. In this way, the relative atomic velocities and the total kinetic energy of the system remain unchanged.

Tabel S1. Reaction energies calculated with MN15 in different basis sets (kcal/mol).

Reaction	Reaction energies calculated with MN15 in different basis sets (kcal/mol)		
	6-31G(d)	6-31G(d,p)	6-31G++(d,p)
$CH_4 + O_2 \rightarrow CH_3 + HO_2$	59.02	59.06	62.17
$CH_4 + O \rightarrow CH_3 + OH$	-49.94	-50.09	-46.14
$CH_3O \rightarrow CH_2O + H$	29.15	29.87	27.90
$CH_2O + H \rightarrow CHO + H_2$	-11.27	-10.63	-11.61
$CH_2O \rightarrow CHO + H$	97.49	99.83	95.67

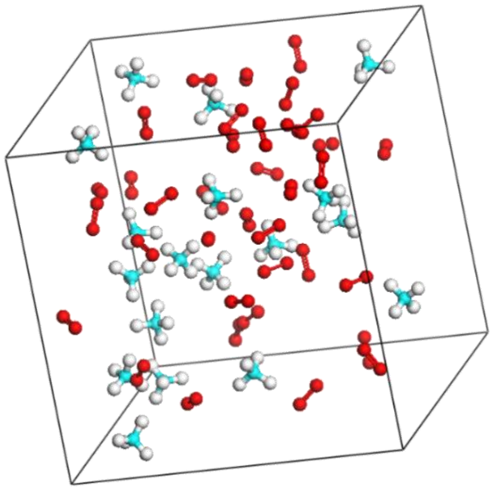

Figure S1. The initial structure of the methane-oxygen system.

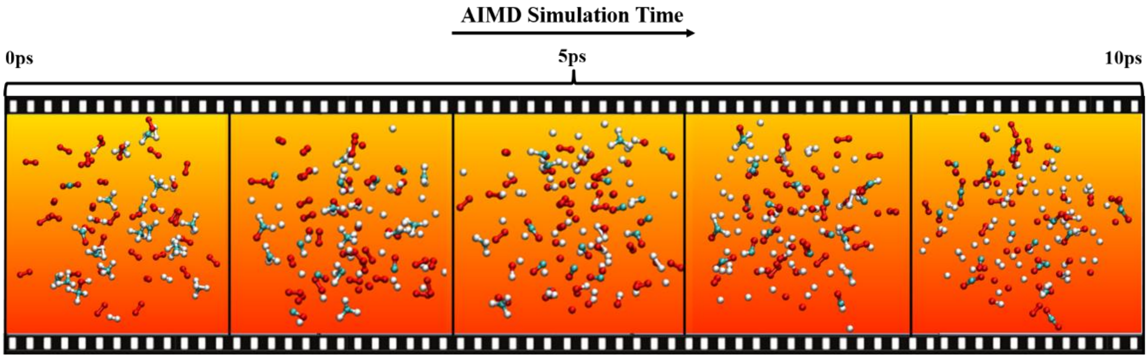

Figure S2. Typical snapshots in the FB-AIMD simulation trajectory. The white, cyan-blue, and red balls represent hydrogen, carbon, and oxygen atoms, respectively.

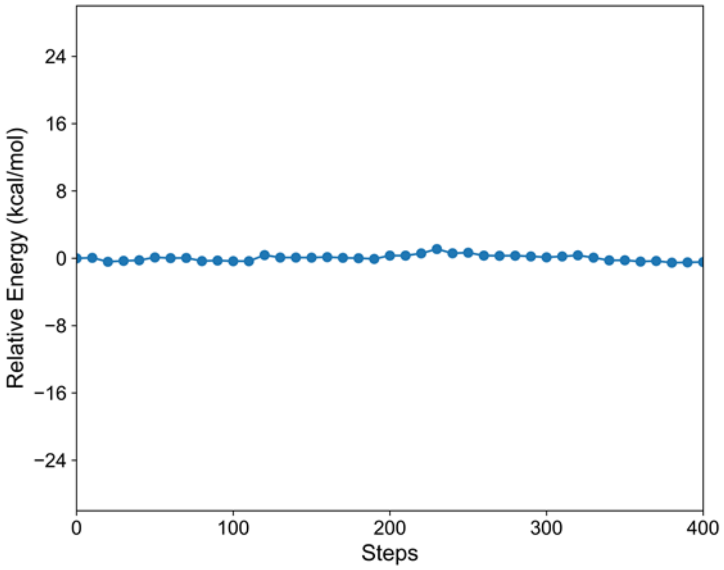

Figure S3. The relative total energy of the system during the simulation under the NVE ensemble.
